# Peripheral Giant Cell Granuloma of the Jaws as First Sign of Primary Hyperparathyroidism: A Case Series

**DOI:** 10.3390/jcm9124042

**Published:** 2020-12-14

**Authors:** Luisa Limongelli, Angela Tempesta, Dorina Lauritano, Eugenio Maiorano, Giuseppe Ingravallo, Gianfranco Favia, Saverio Capodiferro

**Affiliations:** 1 Department of Interdisciplinary Medicine, “Aldo Moro” University of Bari, 70124 Bari, Italy; angela.tempesta1989@gmail.com (A.T.); gianfranco.favia@uniba.it (G.F.); capodiferro.saverio@gmail.com (S.C.); 2 Centre of Neuroscience of Milan, Department of Medicine and Surgery, University of Milano-Bicocca, 20126 Milan, Italy; dorina.lauritano@unimib.it; 3 Department of Emergency and Organ Transplantation, University of Bari Aldo Moro, 70124 Bari, Italy; eugenio.maiorano@uniba.it (E.M.); giuseppe.ingravallo@uniba.it (G.I.)

**Keywords:** perhipheral giant cell granuloma, primary hyperparathyroidism, oral pathology, head and neck pathology

## Abstract

Peripheral giant cell granulomas (PGCG) associated with hyperparathyroidism (HPT) are rare clinical entities. The aim of this study is to report on 21 PGCGs of the oral cavity as the first clinical sign of unknown primary HPT (PHPT) referred to the Complex Operating Unit of Odontostomatology of Aldo Moro University of Bari from 2009 to 2019. Surgical treatment consisted in conservative enucleation of the lesion, if possible, with contextual bone rim osteoplasty with piezosurgical tools and following histological examination. After histological diagnosis of PGCG, PHPT screening was performed dosing parathyroid hormone and serum calcium. In all the patients haematological investigation demonstrated elevated values of parathyroid hormone and serum calcium ruling out an unknown PHPT. Specifically, after endocrinological evaluation, patients showed PHPT related to: parathyroid adenoma (13), parathyroid hyperplasia (two, one of which occurred in a intra-thyroidal parathyroid), and parathyroid carcinoma (1) and were scheduled for surgical treatment. Considering that PGCGs could represent the first clinical sign of an undiagnosed PHPT and the screening of PHPT is a non-invasive and cheap exam, in case of histological diagnosis of a giant cell lesion, both central and peripheral, especially in patients with synchronous or history of methacronous giant cell lesions, parathyroidal screening should be mandatory.

## 1. Introduction

Peripheral giant cell granuloma (PGCG) is a benign, reactive, exophytic gingival lesion that accounts for less than 10% of all gingival lesions [1]. PGCG is usually found in adults with peak in incidence in the age group of 30–40 years [2].

It is believed that the source of PGCG is either the periosteum or periodontal ligament [3].

Clinically, PGCG may present as a firm, soft nodule and as a sessile or pedunculated mass, confined to the alveolar and gingival mucosa [4], often with extension in deeper hard tissues with resorption of the cortical bone [5].

There are various etiological factors for PGCG such as poor oral hygiene, food impaction, previous dental extractions, xerostomia, hormonal imbalance such as hyperparathyroidism (HPT) [1,6].

PGCGs associated with hyperparathyroidism (HPT) are rare clinical entities.

HPT, first described by Von Recklinghausen in 1891, occurs in about 0.05–0.1% of the population with prevalence in middle-age women, with a female to male ratio of 3:1, and is distinguished in primary, secondary and tertiary [7]. Primary HPT (PHPT) occurs in the setting of excessive parathyroid hormone (PTH) secretion by an autonomous gland resulting in hyper-calcemia. Secondary HPT occurs in the setting of hypo-calcemia or vitamin D deficiency acting as a stimulus for PTH production. Tertiary HPT is associated with renal failure and results from autonomous functioning glands in patients with long-standing secondary HPT [8].

The clinical presentation of PHPT varies from asymptomatic disease (seen in countries where biochemical screening is routine) to classic symptomatic disease in which renal and/or skeletal complications are observed [9].

The aim of this retrospective study is to report on 21 PGCGs of the oral cavity as the first clinical sign of unknown PHPT referred to the complex operating unit of odontostomatology of Aldo Moro University of Bari from 2009 to 2019, giving a diagnostic-therapeutical protocol for lesions with histological diagnosis of PGCG.

## 2. Materials and Methods

This study was carried out in accordance to the principles of the Declaration of Helsinki and approved by the independent ethical committee active in the University of Bari, Italy (Study no. 4599, Prot. 1528/C.E.); patients released informed consent for diagnostic and therapeutic procedures and for the possible use of the biologic samples for research purposes.

Patients included in the current retrospective study followed these inclusion criteria:

- Were referred to Complex Operating Unit of Odontostomatology, Aldo Moro University of Bari, from 2009 to 2019;
- Histological diagnosis of PGCG;
- Unknown primary hyperparathyroidism diagnosed by parathyroid hormone dosage after PGCGs surgical removal.

During general anamnesis, the following data were collected: age and gender, other diseases, pharmacologic therapies and previous surgical removals of PGCGs with histological diagnosis before 2009 were also investigated.

The following clinical data were collected: features of the lesion (site, size, number, association with teeth or implants, bleeding), quality of oral hygiene, Plaque Index (by Silness and Loe) for teeth or implants involved in the lesions, referred symptoms. Panoramic radiographs were performed in all instance associated with cone beam computed tomographies if necessary, revealing resorption of lamina dura and thickening of periodontal ligament of involved teeth.

Before surgery, all the patients underwent full mouth disinfection using piezoelectric tools in order to remove irritating factors. Surgical treatment consisted in conservative enucleation of the lesion, if possible with contextual bone rim osteotomy, followed by osteoplasty with piezosurgical tools, and histological examination. The removal of involved teeth/implants was evaluated in each case considering the severity of bone resorption and teeth/implants mobility. The patients were prescribed with antibiotics and analgesics, and oral hygiene instructions were given.

The whole surgical samples were promptly fixed in neutral-buffered formalin for 48 h and sent to the Pathological Anatomy Unit of the University of Bari, embedded in paraffin, sectioned at 4-μm thickness, and stained with haematoxylin-eosin (H&E—Cosmos Biomedical, Swadlincote, UK).

After histological diagnosis of PGCG, HPT screening was performed by dosage of parathyroid hormone and serum calcium. In cases of parathyroid hormone increase, patients were sent to endocrinologist for parathyroid assessment that consisted variably in ultrasound analysis, scintigraphy of parathyroid glands with Technetium-99, MRI and eco-guided Fine Needle Aspiration Citology (FNAC) if necessary.

Patients underwent clinical and radiological follow up.

## 3. Results

From a pool of 192 patients treated for PGCG from 2009 to 2019, sixteen were enrolled in this retrospective study, 11 females and 5 males (M/F ratio 2:1) 57 (±2) years old on average. General medical history was not significant in all instances except for one female patient affected by Hashimoto thyroiditis. Four patients (25%—1 male and 3 females) referred a previous surgical removal of an epulis-like lesion with histological diagnosis of giant cell lesion. Because of previously treated lesions were located in different oral site, the new ones were not considered recurrences. Because of 5 patients (31%) presented two synchronous lesions, totally 21 PGCG were treated.

**Figure 1:**
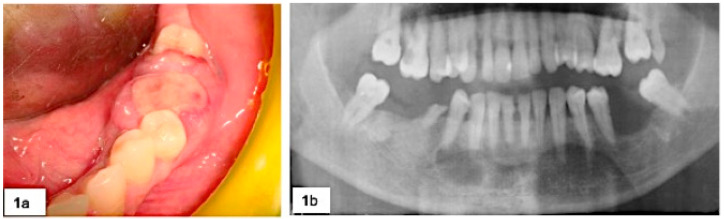
(**a**) Rapidly growing lesion in 3.6 region with severe horizontal bone loss and mobility of both 3.5 and 3.7 (**b**).

All the lesions were reddish-bluish, firm, painless and rapidly growing. Seventeen were sessile while 4 were peduncolated; 18 lesions were ulcerated and spontaneously bleeding (Figure 1). Clinical data of lesions were resumed in Table 1.

**Figure 2:**
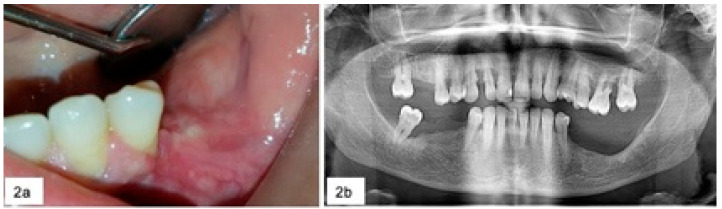
Clinical (**a**) and radiological (**b**) follow-up of the patient after 18 months.

During surgery, in the five cases of severe horizontal bone loss, five teeth and one implant were removed (Figure 2) and abundant bleeding was noticed in 18 cases.

**Figure 3:**
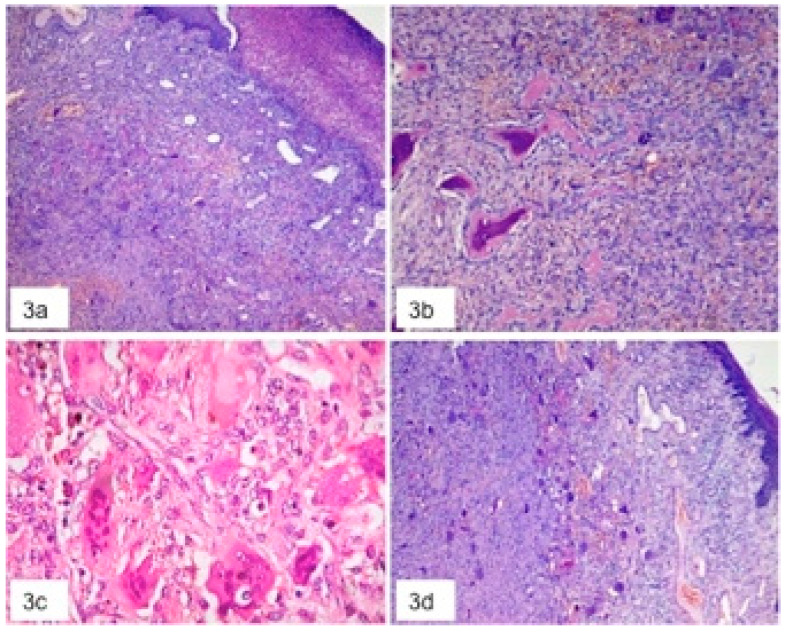
Histological aspects of peripheral giant cell granulomas (PGCG) with giant cells and stromal new formed osteoid bone. (**a**) Panoramic view of PGCG with ulcerated covering mucosa in the upper right region; (**b**) Thin trabecular osteoid bone with surrounding cellular stroma with spindle-shaped fibroblast-like cells (**c**) 40× magnification showing giant cells with variable number of nuclei and different shapes (**d**) Panoramic view with many giant cells surrounded by stroma.

In all instances, the histological examination revealed a great number of multinucleated giant cells of varying sizes and shapes, composed of 2–10 nuclei with a surrounding basophilic stroma rich of mononuclear spindle shaped cells, with numerous and various size of thin walls vascular structures mainly with capillary and venular aspects, focally intermingled with small size arteries; inflammatory infiltrate, haemorrhage, deposit of hemosiderin and scattered areas of thin trabecular osteoid bone were detectable (Figure 3). The final diagnosis was PGCG in all instances.

**Figure 4:**
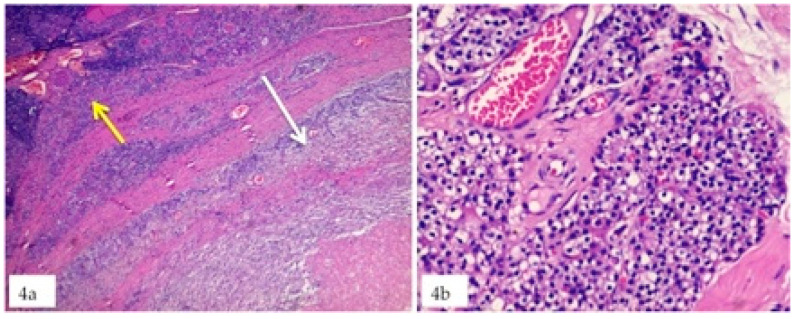
(**a**) Thyroid gland with Hashimoto thyroiditis (yellow arrow) and hyperplasia of an intra-thyroidal Parathyroid (withe arrow); (**b**), 40× magnification showing parathyroid gland hyperplasia with clear and chief aspects of the cells.

In all the patients enrolled in this study, haematological investigation demonstrated elevated values of parathyroid hormone and serum calcium ruling out an unknown primary HPT. Specifically, after endocrinological evaluation, non-invasive radiological assessment of parathyroid glands and eco-guided FNAC, 13 patients showed HPT related to parathyroid adenoma, 2 related to parathyroid hyperplasia (one of which occurred in a intra-thyroidal parathyroid—Figure 4), and 1 related to parathyroid carcinoma and were scheduled for surgical treatment.

No recurrence was noticed except for two patients with PHPT, one female with parathyroid hyperplasia that refused surgical treatment and one male with adenoma with delaying in surgical treatment.

## 4. Discussion

In Europe, PHPT is identified as a relatively common, asymptomatic disorder affecting 21/1000 women aged 55–75 years [9,10].

The prevalence of PHPT associated with giant cell lesions is 5.9% [7].

Peripheral giant cell granuloma (PGCG) is a non-neoplastic lesion of the oral mucosa arising on the buccal or lingual attached gingiva or alveolar mucosa and the crest of the edentulous alveolar ridge [5].

Peripheral manifestation of giant cell lesions in the oral cavity related to PHPT is considered rare and often can occur in an early stage of an unknown primary hyperparathyroidism [11]. The authors in this retrospective study presented 21 PGCGs occurred in 16 patients as first clinical sign of unknown primary HPT.

The occurrence of both PGCG and PHPT is more in females [12]. The ratio of female and male for PGCC is 2:1 and for PHPT is 3:1 [13,14]. Moreover, in this study female predilection is shown; posterior mandible is also the main occurring site accounting for 67% of the lesions. Female predilection cloud be related to positive influence of oestrogen and progesterone [1].

PGCG can arise from periodontal ligament or periosteum of the alveolar bone [15]. While periodontal ligament could be involved in the genesis of PGCGs that arise around teeth, periosteum and cortical bone are certainly involved in the cases in which PGCGs arises on edentulous region or around implants. The exact origin of giant cells is uncertain but it has been suggested that cells like osteoblasts, macrophages and also all the monocyte family, and endothelial cells, and spindle cells can give rise to these multinucleated giant cells [15,16].

There are various etiological factors for PGCG such as poor oral hygiene, food impaction, xerostomia, pregnancy, hormonal imbalance, and hyperparathyroidism (HPT) [1,17]. In cases of multiple lesions, both synchronous and metachronous, once removed classic irritating factors, clinicians should suspect hormonal imbalances.

Clinically, it appears as a sessile or broad-based pedunculated lesion, bluish to purple-red, fleshy or firm swelling with a frequently ulcerated surface [18,19,20].

Ulceration and bleeding are frequent after chewing trauma, especially in bigger lesions.

Radiographic appearance could range from minimal/absent bone resorption to severe horizontal bone loss. Flaitz described resorption of the bone underlying the lesion as corresponding to areas of cortical flattening in edentulous areas and interdental bone loss in dentate patients [21]. In contrast, other authors considered that such bone loss could be the cause rather than the result of PGCG [22].

In this study the majority of patients presented horizontal bone loss, mild in 13 cases and severe in 5. In case of severe bone loss, it is difficult to understand if the giant cell lesion originates in the trabecular hard tissue extending in the periodontal region or if it arises from deeper tissues with secondary superficial spreading. Probably the clinical history and the analysis of previous radiological exams could resolve the dilemma.

The treatment of the PGCG is complete excision of the lesion along with the curettage of the base and borders of the lesion [23]. The recurrence rate of the lesion is 5.0–70.6% [1].

This wide range and high recurrence rate could be related both to incomplete removal of the lesions along with teeth or implants severely involved in the lesions and probably to the lack of screening of hormonal imbalances after certain histological diagnosis of PGCG is achieved. Histologically, PGCG not associated with PHPT are characterized by an increase of microvessel density and Bcl-2 and CD68 expressions [24] and mutations of KRAS and MAPK [25]. Nevertheless, further studies are necessary in order to understand if these findings are detectable in PGCG associated to PHPT too.

Considering the possibility that PGCGs could represent the first clinical sign of an undiagnosed PHPT and the fact that the screening of PHPT is a non-invasive and cheap exam, already used in some countries, in case of histological diagnosis of a giant cell lesion, both central [26] and peripheral, especially in patients with synchronous or history of methacronous giant cell lesions, should be mandatory parathyroidal screening.

## Figures and Tables

**Table 1:** Clinical and radiological data of the lesions.

Lesions Data	
Site	*n*
Posterior lower jaw periodontal tissue	14 (66.6%)
Anterior lower jaw periodontal tissue	2 (9.6%)
Posterior upper jaw periodontal tissue	4 (19%)
Anterior upper jaw periodontal tissue	1 (4.8%)
Size	*n*
<2 cm	12 (57.1%)
>2 cm	9 (42.9%)
Plaque index (By Silness and Loe)	*n*
Poor	11 (52.4%)
Fair	6 (28.5%)
Good	3 (14.3%)
Excellent	1 (4.8%)
Involved teeth/implants	*n*
Teeth	17 (80.9%)
Implants	4 (19.1%)
Bone resorption	*n*
No	3 (14.3%)
Mild horizontal bone loss (<3 mm)	13 (61.9%)
Severe horizontal bone loss (>3 mm)	5 (23.8%)
Symptoms	*n*
Pain	24%
Chewing difficulties	80%
Tooth/implant mobility	12%
Bleeding	86%
Median time of presentation	4 months
